# Environmental Geochemistry of Cerium: Applications and Toxicology of Cerium Oxide Nanoparticles

**DOI:** 10.3390/ijerph120201253

**Published:** 2015-01-23

**Authors:** Jessica T. Dahle, Yuji Arai

**Affiliations:** 1 School of Agricultural, Forest and Environmental Science, Clemson University, Clemson, SC 29634, USA; E-Mail: dahle.jessica@gmail.com; 2 Department of Natural Resources and Environmental Sciences, University of Illinois at Urbana-Champaign Urbana, IL 61801, USA

**Keywords:** cerium, cerium oxide, nanoparticles, toxicity, fate, geochemistry, lanthanide

## Abstract

Cerium is the most abundant of rare-earth metals found in the Earth’s crust. Several Ce-carbonate, -phosphate, -silicate, and -(hydr)oxide minerals have been historically mined and processed for pharmaceutical uses and industrial applications. Of all Ce minerals, cerium dioxide has received much attention in the global nanotechnology market due to their useful applications for catalysts, fuel cells, and fuel additives. A recent mass flow modeling study predicted that a major source of CeO_2_ nanoparticles from industrial processing plants (e.g., electronics and optics manufactures) is likely to reach the terrestrial environment such as landfills and soils. The environmental fate of CeO_2_ nanoparticles is highly dependent on its physcochemical properties in low temperature geochemical environment. Though there are needs in improving the analytical method in detecting/quantifying CeO_2_ nanoparticles in different environmental media, it is clear that aquatic and terrestrial organisms have been exposed to CeO_2_ NPs, potentially yielding in negative impact on human and ecosystem health. Interestingly, there has been contradicting reports about the toxicological effects of CeO_2_ nanoparticles, acting as either an antioxidant or reactive oxygen species production-inducing agent). This poses a challenge in future regulations for the CeO_2_ nanoparticle application and the risk assessment in the environment.

## 1. Introduction

Cerium (Ce) is a member of the lanthanide series of metals on the periodic table. The discovery of Ce in an oxide form was first reported in 1803 by scientists in both Sweden and Germany [1]. This oxide was given the name “ceria” by discoverer Jons Jacob Berzelius in Sweden. Berzelius named the compound after the dwarf planet Ceres, which itself was named for the Roman goddess of agriculture. Cerium is the most abundant of the rare-earth metals found in the Earth’s crust [2], where it comprises about 0.0046% by weight. Cerium and other lanthanide metals naturally occur in an array of minerals in the environment including carbonates (e.g., bastnäsite, (Ce,La)(CO_3_)F), silicates (e.g., allanite, (Ca,Ce)(Al_2_Fe^2+^)(Si_2_O_7_)(SiO_4_)O(OH)), cerite (Ce,Ca)_9_(Fe,Mg)(SiO_4_)_3_(HSiO_4_)_4_(OH)_3_), phosphates (e.g., monazite,(Ce,La,Nd,Th)(PO_4_)), and oxides (e.g., euxenite, (Y,Ca,Ce,U,Th) (Nb,Ti,Ta)_2_O_6_)). Of these minerals, however, only bastnäsite and monazite have been serving a major role for commercial uses. In the last several years, Ce-based compounds have received much attention because of their popular applications of CeO_2_ nanoparticles (NPs) in industrial applications. Unfortunately, due to the relative newness of CeO_2_ NP environmental chemistry as a field, the impact of CeO_2_ NPs on ecosystem health (e.g., toxicological effects on aquatic-terrestrial organisms) has not been well understood. This review (discusses the geochemistry of Ce, geological occurrence, its applications and uses, environmental fate and the contradicting findings in recent toxicological studies.

## 2. Cerium Chemistry

### 2.1. Aqueous Geochemistry

Nearly 89% of naturally occurring Ce consists of four observationally stable isotopes (^136^Ce, ^138^Ce, ^140^Ce, ^142^Ce). With the exception of ^140^Ce (the most abundant isotope at 88.5% natural occurrence), these isotopes are expected to undergo double beta decay. Cerium (electronic configuration: [Xe] 4f^1^ 5d^1^ 6s^2^) exhibits a variable electronic structure which sets it apart from other lanthanides. The energy of the inner 4f level is nearly equal to the energy of outer or valence electrons. Since a very small amount of energy is necessary to change the relative occupancy (of these electronic levels), dual valence states of +3 and +4 occur in cerium [3]. In low temperature geochemical environments, cerium commonly exists in both the trivalent cerous state (Ce^3+^) and the tetravalent ceric state (Ce^4+^). Cerium exhibits a unique stability in the tetravalent state; other lanthanides are only stable in the trivalent state. This feature allows for easy separation of cerium from other rare-earth elements through oxidation (forming CeO_2_) followed by variable solubility filtration.

The formation of Ce aqueous complexes and solubility products involve the binding of a metal center (like Ce^3+^ or Ce^4+^) to a ligand to form a coordination complex. These interactions are normally soft-soft or hard-hard as described below by the hard and soft acid and base (HSAB) theory. Hard-hard affinity can be described as mostly ionic while soft-soft affinity is covalency-driven. Characterizing the electronegativity, size, and charge properties of cerium is key to understanding its association chemistry since the affinity of metals for ligands is controlled by these three factors.

The HSAB theory can be used to explain the tendency of Ce^3+^ to quickly and strongly bond with ligands such as phosphates and hydroxides over species like sulfates and hydrides (Table 1). Using HSAB, species are first characterized as a Lewis acid or Lewis base, then identified as a “hard” or “soft” species. Hard species exhibit a small atomic/ionic radius, high oxidation state, and are only weakly polarizable while soft species are large in size, have low oxidation states, and are strongly polarizable. Common examples of hard ions include Ca^2+^, Fe^3+^ and OH^−^, Ag^+^, Cd^2+^ and R-SH^−^ are some well-known soft ions. Hard acids have a strong affinity for hard bases while soft acids “prefer” to bond with soft bases. Intermediate ions, such as Pb^2+^, that have no strong preference for hard over soft bonding partners also exist. Cerium is classified as a hard acid based on its small size, high oxidation state (most commonly 3^+^ or 4^+^) and weak polarizability; thus, it is most likely to bond with hard bases. Hard bases commonly found in aquatic and terrestrial environment are OH^−^, F^−^, PO_4_^3−^, SO_4_^2−^, Cl^−^, CO_3_^2−^, ClO_4_^−^ and NO_3_^−^. Cerium(3+) does not preferably react with soft bases including I^−^, CN-, CO, S^2−^, sulfhydryl (R-SH), and R-S^−^ when sufficient supplies of hard bases are available in the system.

**Table 1:** Distribution of Lewis acids and Bases of common metals and ligands in the low-temperature geochemical environment (Modified after [4]).

	Acids	Bases
**Hard**	H^+^, Li^+^, Na^+^, K^+^ Mg^2+^, Ca^2+^, Sr^2+^, Ce^3+^	NH_3_, R-NH_2_, H_2_O, OH^−^, O^2−^, CH_3_COO^−^, CO_3_^2−^, NO_3_^−^, PO_3_^4−^, SO_4_^2−^, F^−^
**Intermediate**	Fe ^2+^, Co^2+^, Ni^2+^, Cu^2+^, Zn^2+^, Pb^2+^	NO_2_^−^, SO_3_^2−^, Br^−^
**Soft**	Cu^+^, Ag^+^, Cd^2+^+, Hg^+^, Hg^2+^	CN-, CO, S^2−^, R-SH, R-S^−^

Cerium oxide is most often noted for their catalytic ability, which is largely affected by redox behavior and is known to be directly dependent on cerium’s redox state. The nanoparticles are often largely composed of Ce(III) (up to 60% in some cases) as well as Ce(IV); the more Ce(IV) in the nanoparticles, the stronger the catalase mimetic activity [5]. Another study has shown that a decrease in the 3^+^/4^+^ ratio directly correlates with a loss of superoxide dismutase (SOD) mimetic activity [6]. Clearly, redox state plays a large role in determining the characteristics and behavior of cerium oxide nanoparticles.

The primary hydration numbers of most lanthanides are 8 and 9; salts with common anions frequently contain Ln(H_2_O)_9_^3+^ with tri-capped trigonal prismatic geometry. Cerium’s normal coordination/hydration number of 6 is quite uncommon among lanthanides. However, as previously stated, cerium does exhibit other coordination numbers as well; for example, in the case of nitrates, Ce(NO_3_)_5_^2−^ features a 10-coordinate bicapped dodecahedron structure and Ce(NO_3_)_6_^3−^ features a 12-coordinate icosahedron structure. Other common O-donor chelating ligands that form complexes with Ce and other lanthanides include oxalate, citrate, tartrate, and beta-diketonates. Classic bidentate complexes, macrocyclic ligands (crown ethers), and weak coordination by halides are also key to the solution chemistry of 3^+^ lanthanides [7].

In terms of 4^+^ lanthanides, cerium is the only tetravalent lanthanide with significant coordination and/or aqueous chemistry. Lower oxidation states occur in bromides and iodides while higher oxidation states of lanthanides tend to occur in fluorides and oxides. The E° value of Ce(IV)aq/Ce(III)aq = 1.72 V; the E° value of other lanthanides is ~2.9 V. This value is largely dependent on hydrolysis and complexation properties of Ce including pH. In solution, hydrolysis to Ce(OH)^3+^ occurs, then polymerization which leads to precipitation of yellow gelatinous hydrated Ce(IV) oxide [7].

### 2.2. Solubility and Chemical Properties of Ce Compounds

The solubility properties of Ce compounds such as these listed in Table 2 vary greatly. Solubility constants for Ce(III) and Ce(IV) bound to common ligands including hard bases like phosphate are listed in Table 1. Cerium phosphate readily precipitates out and is extremely insoluble. Cerium(IV) hydroxide exhibits similar properties. However, Ce(III) hydroxide is many orders of magnitude more soluble than cerium(IV) hydroxide. Among cerium species, even Ce(III) hydroxide is not regarded as particularly soluble. Among the most soluble species are Ce(III) chloride and cerium(III) nitrate. Cerium(III) selenite and cerium(III) sulfate are also relatively soluble.

**Table 2:** Solubility and solubility constants for selected Ce compounds [8,9,10,11,12,13] Units are in grams per 100 g H_2_O unless otherwise mention in the table.

Species	K_sp_°
CeO_2_ bulk	0.00073338
CeO_2_ NP	0.128205128
Ce(OH)_3_	1.6E−20
Ce(OH)_4_	2.00E−48
CePO_4_	1.00E−23
Ce_2_S_3_	6.00E−11
Ce_2_(SO_4_)_3_·2H_2_O	9.84 (L^3^∙mol^−2^)
Ce(SO_4_)_2_	9.84 (L^2^∙mol^−2^)
CeF_3_	8.00E−16
Ce(IO_3_)_3_	3.20E−10
Ce(IO_3_)_4_	5.00E−17
Ce_2_(SeO_3_)_3_	3.70E−25
Ce(NO_3_)_3_	234 (L^2^∙mol^−2^)

Analogues of Ce, or lanthanides and possibly actinides that react similarly and become more soluble as the valence state becomes more reduced, are difficult to identify since the tetravalent state is only characteristic of cerium. Since it is not possible to identify “true” analogues, instead comparisons must be made across systems with 3^+^/2^+^ systems (such as that of europium) instead of cerium’s 4^+^/3^+^ system. Cerium has the notable characteristic of becoming more soluble as its coordination state becomes more reduced. Other lanthanides are only stable at 3^+^ with the exception of europium (Eu), which may be the only “good” analogue of Ce in the lanthanides. Europium usually assumes a trivalent oxidation state, but a divalent oxidation state is also very common. The solubility constant of Eu(III) hydroxide at 20 °C is 1.538 × 10^−5^, which is similar in magnitude but overall less soluble than Ce(III) hydroxide. The solubility constant of Eu(III) sulfate at 20 °C is 2.56, which is also much less soluble than Ce(III) sulfate. The sulfate of Eu(II) is highly insoluble in water. Other compounds of Eu(III) exhibit similar properties such that the compounds of Eu(III) tend to be far less soluble than the same compounds of Ce(III). Like the Eu(III) compounds discussed above, most lanthanides existing in the trivalent state are not very soluble in water. The lighter lanthanides are more soluble when paired with oxalates, double sulfates, or double nitrates, while the heavier lanthanides are more soluble with basic nitrates.

The redox state of Ce in CeO_2_ NPs not only affects the solubility of Ce compounds; it also has implications for industrial applications, particularly for CeO_2_ catalyst. Cerium oxide nanoparticles are most often noted for their catalytic ability, which is known to be directly dependent on cerium’s redox state. The nanoparticles are often largely composed of Ce(III) (up to 60% in some cases) as well as Ce(IV); the more Ce(IV) in the nanoparticles, the stronger the catalase mimetic activity [5]. Another study has shown that a decrease in the 3^+^/4^+^ ratio directly correlates with a loss of SOD mimetic activity [6]. Clearly, redox state plays a large role in determining the characteristics and behavior of CeO_2_ NPs. A UV absorbing/filtering property of Ce(IV)O_2_ is recently adapted in (un)doped coatings of industrial products and sunscreen [14]. Under UV, an electron can be transferred to the conduction band creating a hole in the valence band. In the case of CeO_2_ NPs, they show a very fast recombination of charge carriers, resulting in no further creation of free radicals. This makes CeO_2_ a good UV absorber.

As noted earlier, the variable electronic structure of cerium (electronic configuration: [Xe] 4f^1^ 5d^1^ 6s^2^) truly sets it apart from other lanthanides. The energy of the inner 4f level is nearly equal to the energy of outer (valence) electrons. Since a very small amount of energy is necessary to change the relative occupancy (of these electronic levels), dual valence states of +3 and +4 occur in cerium [3]. The electronegativity value of Ce is 1.12 on the Pauling scale; the electronegativity values of other common species found in soils are shown in Table 3. Cerium has three ionization energies. Its first ionization energy is 534.4 kJ∙mol^−1^ (Ce(IV)), its second ionization energy is 1050 kJ·mol^−1^ (Ce(III)), and its third ionization energy is 1949 kJ∙mol^−1^ (Ce(II)).

**Table 3:** Electronegativity of cerium (Pauling scale) compared to other common elements found in soils [15].

Species	EN	Species	EN	Species	EN
Ar	0.4	Al	1.61	P	2.19
K	0.82	Zn	1.65	Pb	2.33
Na	0.93	Cd	1.69	C	2.55
Sr	0.95	Co	1.88	S	2.58
Ca	1	Si	1.9	N	3.04
Ce	1.12	Cu	1.9	Cl	3.16
Mg	1.31	Ni	1.91	O	3.44

Using Pauling’s rules, coordination properties of Ce compounds can be predicted. The coordination number of Ce(IV)oxide (perhaps the most environmentally relevant Ce species) is 6, so the crystal exhibits an octahedron structure. Cerium(III) oxide also features a coordination number of 6 and the same octahedron crystal structure. However, cerium compounds are not always coordinated octahedrally; for the compounds CeF_6_^2−^ (via fluoride bridging) and CeCl_6_^2−^, the coordination number is 8. The tetravalent charge seems to stabilizes halogeno-complexes like CeF_8_^4−^ [7].

## 3. Geological Occurrence

Cerium is found in a variety of mineral classes, primarily including carbonates, phosphates, silicates, oxides and hydroxides. Main sources of industrial cerium include the carbonate mineral bastnäsite and the phosphate mineral monazite. These key cerium minerals and other lanthanide associated minerals are discussed in detail below.

### 3.1. Carbonates

Carbonates, the largest group of Ce-containing minerals, can be grouped in anhydrous, hydroxyl or halogen, and hydrated groups (Table 4). Some of anhydrous carbonates are burbankite, carbocernaite, remondite, sahamalite, and tundrite. Carbonates with hydroxyl or halogen are bastnäsit, daqingshanite, huanghoite, hydroxylbastnäsite, parasite, rontgenite, and synchysite. Hydrate carbonates are ancylit, calcioancylite, lanthanit, rhabdophane, and thorbastnäsite.

**Table 4:** Several members of the rare earth anhydrous and hydrous carbonates. Modified after [16].

Mineral	Chemical Formula	Type Locality
**Anhydrous**		
Bastnäsite-(Ce)	$(\text{Ce},\text{La})({\text{CO}}_{3})\text{F}$	Sweden
Burbankite	${\left(\text{Na},\text{Ca}\right)}_{3}{\left(\text{Sr},\text{Ba},\text{Ce}\right)}_{3}{\left({\text{CO}}_{3}\right)}_{5}$	USA
Carbocernaite	$(\text{Ca},\text{Na})(\text{Sr},\text{Ce},\text{Ba}){\left({\text{CO}}_{3}\right)}_{2}$	Russia
Cordylite-(Ce)	$(\text{Na},{\text{BaCe}}_{2}{\left({\text{CO}}_{3}\right)}_{4}\text{F}$	Greenland
Huanghoite	$\text{BaCe}{\left({\text{CO}}_{3}\right)}_{2}\text{F}$	China
Hydroxylbastnäsite-(Ce)	$\left(\text{Ce},\text{La}){\left(\text{CO}\right)}_{3}(\text{OH},\text{F}\right)$	Russia
Parisite-(Ce)	$\text{Ca}{\left(\text{Ce},\text{La}\right)}_{2}{\left({\text{CO}}_{3}\right)}_{3}{\text{F}}_{2}$	Colombia
Remondite-(Ce)	${\text{Na}}_{3}{\left(\text{Ce},\text{La},\text{Ca},\text{Na},\text{Sr}\right)}_{3}{\left({\text{CO}}_{3}\right)}_{5}$	Cameroon
Rontgenite	${\text{Ca}}_{2}{\left(\text{Ce},\text{La}\right)}_{3}{\left({\text{CO}}_{3}\right)}_{5}{\text{F}}_{3}$	Greenland
Sahamalite	$(\text{Mg},\text{Fe}){\left(\text{Ce},\text{La},\text{Nd}\right)}_{2}{\left({\text{CO}}_{3}\right)}_{4}$	USA
Synchysite-(Ce)	$\text{Ca}(\text{Ce},\text{La}){\left({\text{CO}}_{3}\right)}_{2}$F	Greenland
**Hydrous**		
Ancylite-(Ce)	$(\text{SrCe}{\left({\text{CO}}_{3}\right)}_{2}\left(\text{OH}\right)\cdot {\text{H}}_{2}\text{O}$	Greenland
Calcioancylite	$(\text{Ca},\text{Sr})\text{Ce}{\left({\text{CO}}_{3}\right)}_{2}\left(\text{OH}\right)\cdot {\text{H}}_{2}\text{O}$	Russia
Calkinsite	${\left(\text{Ce},\text{La}\right)}_{2}{\left({\text{CO}}_{3}\right)}_{3}\cdot 4{\text{H}}_{2}\text{O}$	USA
Daqingshanite-(Ce)	${\left(\text{Sr},\text{Ca},\text{Ba}\right)}_{3}(\text{Ce},\text{La}){\left({\text{CO}}_{3}\right)}_{3–\text{x}}({\text{PO}}_{4})){\left(\text{OH},\text{F}\right)}_{2\text{x}}$	China
Lanthanite-(Ce)	${\left(\text{Ce},\text{La},\text{Nd}\right)}_{2}{\left({\text{CO}}_{3}\right)}_{3}\cdot 8{\text{H}}_{2}\text{O}$	Wales
Rhabdophane	$(\text{Ce},\text{La})({\text{PO}}_{4})\cdot {\text{H}}_{2}\text{O}$	England
Thorbastnäsite	$\text{Th}(\text{Ca},\text{Ce}){\left({\text{CO}}_{3}\right)}_{2}{\text{F}}_{2}\cdot 3{\text{H}}_{2}\text{O}$	Russia
Tundrite-(Ce)	${\text{Na}}_{2}{\text{Ce}}_{2}\text{Ti}({\text{SiO}}_{4}){\left({\text{CO}}_{3}\right)}_{2}{\text{O}}_{2}$	Russia

As stated earlier, bastnäsite (Ce,La)(CO_3_)F) is one of two most important mineral sources of cerium and other rare-earth metals (such as Th). Bastnäsite is of particular importance to the discovery of Ce. An early mining of bastnäsite began at the Mountain Pass rare earth mine in San Bernardino, California with the discovery of a new class of rare earth deposit: a large carbonaceous mineral deposit containing significant amounts of bastnäsite. The major composition of bastnäsite consisted of approximately 49% cerium, 33% lanthanum, 12% neodymium with some praseodymium, samarium and gadolinium. Bastnäsite minerals also exhibited at least twice the Eu concentrations of a typical monazite. As more carbonatite deposits were discovered in Africa and China, mining processes were developed and these deposits became vital to the global lanthanide supply. From 1965 to the mid- 1980s, the Mountain Pass site emerged as the world’s major source of lanthanides. Currently China plays a leading role in the global rare earth supply (as high as 97%); the Bayan Obo Mining District is the source of the majority of the world’s lanthanides [16].

### 3.2. Phosphates

Though relatively few cerium phosphate minerals exist, they are very important to consider when identifying sources of cerium (Table 5). Until bastnäsite began to be processed around 1965, the phosphate mineral monazite, which usually forms small reddish-brown crystals, was the only significant commercial source of lanthanides such as cerium and thorium. Monazite is most often found in placer deposits, which are mineral accumulations often in the form of black sedimentary rocks. Monazite is fairly dense (about 4.6 to 5.7 g/cm^3^), a property consistent with that of most black sand minerals such as zircon and cassiterite. Placer deposits in India are particularly rich in monazite as well as deposits in Australia, Madagascar and South Africa [17].

**Table 5:** Several members of the rare earth phosphates. Modified after [17].

Mineral	Chemical Formula	Type Locality
Agardite-(Ce)	${\text{CeCu}}_{6}({\left({\text{AsO}}_{4}\right)}_{3}{\left(\text{OH}\right)}_{2}\cdot \cdot 3{\text{H}}_{2}\text{O}$	Germany
Monazite-(Ce)	$(\text{Ce},\text{La},\text{Nd},\text{Th})({\text{PO}}_{2}$)	Russia

One of the major reasons that bastnäsite came to replace monazite in lanthanide production in the 1960’s is the radioactive nature of monazite, which contains Th and produces radioactive daughter products. This radioactive character is useful because it allows monazite to be used as a geological dating tool; thorium was also considered as a potential nuclear fuel in the 1960s. When this interest began to decline, bastnäsite was an attractive replacement option because it contained much lower concentrations of Th than were found in monazite. Monazite is also more difficult to store than bastnäsite since it must be kept away from any mineral samples that are sensitive to radiation.

Monazite and bastnäsite minerals are both broken down into further classifications based on relative elemental composition. Four types of monazite and three types of bastnäsite are commonly referred to in literature. Monazite occurs in monazite-(Ce) [meaning Ce is the most predominant rare earth metal in monazite-(Ce)( (Ce,La,Nd,Th)(PO_4_)], monazite-(La), monazite-(Nd), and monazite-(Pr); bastnäsite occurs in bastnäsite-(Ce), bastnäsite-(La), and bastnäsite-(Y). The –(Ce) compositions constitute most of the bastnäsite and monazite minerals in existence [17].

### 3.3. Silicates

Silicates comprise the most abundant group of minerals in the earth’s crust. Silicates play an extremely important role in soil minerals (Table 6). A significant amount of Ce is found in the silicate mineral allanite,(Ca,Ce,Y)_2_(AlFe^3+^)_3_(SiO_4_)_3_(OH), Cerite-(Ce), (Ce,Ca)_9_(Fe,Mg)(SiO_4_)_3_(HSiO_4_)_4_(OH)_3_, gadolinite-(Ce) (Ce,La,Nd,Y)_2_Fe^2+^Be_2_Si_2_O_10_ and Joaquinite-(Ce), NaBa_2_Ce_2_FeTi_2_[Si_4_O_12_]_2_O_2_(OH,F)·H_2_O.

**Table 6:** Several members of the rare earth silicates. Modified after [17].

Mineral	Chemical Formula	Type Locality
Allanite-(Ce)	$\left(\text{CaCe})({\text{Al}}_{6}{\text{Fe}}^{2+})({\text{Si}}_{2}{\text{O}}_{3})({\text{SiO}}_{4})\text{O}(\text{OH}\right)$	Greenland
Cerite-(Ce)	${\left(\text{Ce},\text{Ca}\right)}_{9}(\text{Fe},\text{Mg}){\left({\text{SiO}}_{4}\right)}_{3}{\left({\text{HSiO}}_{4}\right)}_{4}{\left(\text{OH}\right)}_{3}$	Sweden
Gadolinite-(Ce)	${\left(\text{Ce},\text{La},\text{Nd},\text{Y}\right)}_{2}{\text{Fe}}^{2+}{\text{Be}}_{2}{\text{Si}}_{2}{\text{O}}_{10}$	Japan, Norway
Joaquinite-(Ce)	${\text{NaBa}}_{2}{\text{Ce}}_{2}{\text{FeTi}}_{2}{\left[{\text{Si}}_{4}{\text{O}}_{12}\right]}_{2}{\text{O}}_{2}(\text{OH},\text{F})\cdot {\text{H}}_{2}\text{O}$	USA

Like bastnäsite and monazite, allanite can be broken down into three further classifications: allanite-(Ce) ((CaCe)(Al_2_Fe^2+^)(Si_2_O_7_)(SiO_4_)O(OH)), allanite-(La), and allanite-(Y). Allanite is a useful source of rare earth elements because it can contain up to 20% rare earths by composition. The high concentrations of thorium and other radioactive elements in the mineral lead to radiative degradation and structure disruption of allanite and surrounding minerals. The distribution of allanite minerals is widespread with large, high-quality deposits occurring in Greenland (where allanite was discovered), Sweden, Norway, Finland, and Russia. Allanite has hardness values ranging from 5.5–6 Mohs and is found mainly in metamorphic sedimentary and felsic igneous rocks. Allanite is also commonly referred to as orthite.

### 3.4. Oxides and Hydroxides

Cerium (hyr)oxide minerals are least common of all Ce minerals such as aeschynite-(Ce) (Ce,Ca,Fe,Th)(Ti,Nb)_2_(O,OH)_6_, euxenite-(Y) (Y,Ca,Ce,U,Th)(Nb,Ti,Ta)_2_O_6_, and perovskite CaTiO_3_ with Ce varieties (Table 7). Of the few oxides and hydroxides that contribute to commercial cerium sources, euxenite is the most significant oxide phase despite its low composition percent of cerium (~4%). Unlike allanite, monazite and bastnäsite, euxenite exists almost entirely in the euxenite-(Y) form, featuring yttrium as its major component instead of cerium. Like allanite, euxite also exhibits radiation damage to itself and nearby minerals due to high radioactive content. With hardness values varying between 5.5 and 6.5 Mohs, euxenite features comparable to slightly higher hardness than its other cerium-containing counterparts, allanite, monazite, and bastnäsite. Euxenite occurs along with monazite in black sands as well as in granite pegmatites. Euxenite is found primarily in Norway, where it was discovered, as well as in Russia, Brazil, Madagascar, Canada, and the US. Euxenite functions as an ore for many of the other rare earth elements contained in the mineral as well [16].

As discussed in the previous section, Ce is commonly present with other lanthanides and metals forming phosphate and carbonate minerals. The heterogeneity of natural minerals makes the isolation process of pure Ce extremely difficult. Some ores are directly used for metallurgical purposes. Other Ce enriched ores undergo the purification process prior to industrial and commercial uses. The separation of Ce from natural minerals is conducted via oxidation and variable solubility based filtration steps.

In general, the mineral ores are leached with sulfuric acid and then titrated with sodium hydroxide to remove thorium precipitates. This step is followed by precipitation with oxalate to remove to most of rare earth elements (e.g., Ce, La, Th, Nd) as insoluble oxalate forms. These oxalates undergo annealing/oxidation process to produce lanthanide (tri-and tetra-valent state) oxides. Acidification of these mixed oxides by nitric acid makes all of the lanthanide (III) oxides soluble except for Ce(IV)(OH)_4_. Insoluble Ce(IV) hydroxides are separated through the filtration process [3,18]. Once the purity of Ce is improved from the mixed lanthanide/metal oars, a variety of Ce compounds are synthesized for commercial and industrial applications. The historic and current uses of Ce compounds are discussed below.

**Table 7:** Several members of the rare earth oxides and hydroxides. Modified after [16].

Mineral	Chemical Formula	Type Locality
Aeschynite-(Ce)	$(\text{Ce},\text{Ca},\text{Fe},\text{Th}){\left(\text{Ti},\text{Nb}\right)}_{2}{\left(\text{O},\text{OH}\right)}_{6}$	Russia
Euxenite-(Y)	$(\text{Y},\text{Ca},\text{Ce},\text{U},\text{Th}){\left(\text{Nb},\text{Ti},\text{Ta}\right)}_{2}{\text{O}}_{6}$	Norway
Perovskite	CaTiO_3_ with Ce varieties	Russia

## 4. Historical and Current Uses

### 4.1. Drugs and Pharmaceuticals

Cerium compounds have recorded uses in drugs and pharmaceuticals as early as the mid nineteenth century. In 1854, cerium nitrate was first reported to relieve vomiting. Cerium(III) oxalate was administered for many following decades for anti-vomiting effects in cases of sea sickness, gastrointestinal and neurological disorders, and especially in pregnant women. Cerium(III) oxalate was used as an antiemetic until the mid-1950s when it was replaced by the antihistamine meclizine which is still in use today [19].

Trivalent cerium exhibits similar size and bonding properties to Ca^2+^, an extremely biologically important cation. Ce^3+^ can replace Ca^2+^ in biomolecules due to their similar ionic radius, thus Ce^3+^ compounds strongly exhibit the ability to act as anticoagulant, or anti-clotting, agents. Several of the lanthanides, including cerium, are well-known for their anticoagulant properties and have been employed as antithrombic drugs [19].

Cerium compounds are also known in particular for their uses in topical burn treatments due to their bacteriostatic and bactericidal effects. By the end of the 19th century, Ce compounds were used in both human and veterinary medicine; most commonly used were Ce(III) acetate and Ce(III) stearate treatments. Later, studies confirmed the antiseptic effects of cerium(III) chloride, Ce(III) nitrate, and Ce(IV) sulfate and demonstrated particular subsceptibility of both gram-negative and gram-positive bacteria (which tend to coat burn wounds) to their effects [18]. Cerium(III) nitrate in particular is a widely used treatment for burn wounds, exhibiting nearly a 50% reduction in death rate for patients with life-threatening burns when compared to patients who were administered silver nitrate treatments [20].

### 4.2. Mischmetal

While commercial applications of Ce are numerous, it is most commonly used in the form of mischmetal for metallurgical purposes. Mischmetal is an alloy of rare earth metals in various naturally occurring proportions; a typical composition includes approximately 50% Ce and 25% lanthanum, as well as small amounts of Nd and Pr. Mischmetal reacts with impurities found in metals to form solid compounds, thereby reducing the effect of these impurities on the properties of the metal. Mischmetal is used in steel manufacturing to improve shape control, reduce hot shortness, and increase heat and oxidation resistance [21].

The high scavenging capacity of Ce with respect to oxygen and sulfur can be used to improve oxidative resistance of alloys; however, apart from mischmetal, elemental Ce is not used for a wide variety of applications due to its high reactivity and immediate oxidation potential. However, a wide array of applications arises from the combination of Ce with other materials to produce assorted cerium compounds. Chemical companies began selling Ce compounds in the late 1950s for use primarily in mischmetal; since then, the use of Ce compounds for multiple applications has become widespread.

### 4.3. Organic Synthesis Starting Materials

Cerous chloride (CeCl_3_) is used as a catalyst in Friedel-Crafts alkylation reactions as well as a starting material for the preparation of other Ce salts. Anhydrous cerous chloride is generally prepared via thermal treatment of CeCl_3_·7H_2_O. This hydroscopic solid is important in organic syntheses such as Luche reduction and alkylation of ketones for which organolithium reagents cannot be used [22,23].

### 4.4. Scintillation Counters

Cerous bromide is a component of interest in scintillation counters, which measure ionizing radiation. Aqueous solutions of cerous bromide (CeBr_3_·H_2_O) can be prepared by reacting Ce_2_(CO_3_)_3_·H_2_O with HBr. Heating the product with NH_4_Br followed by sublimation of residual NH_4_Br will allow dehydration, yielding in white hygroscopic cerous bromide (CeBr_3_) solids. Well-ordered single crystals may be produced using standard crystal growth methods such as the Bridgman or Czochralski methods [24]. CeBr_3_-doped lanthanum bromide single crystals exhibit superior scintillation properties; applications for CeBr_3_ include security, medical imaging, and geophysics detectors [25]. Undoped single crystals of CeBr_3_ have also shown promise as gamma ray scintillation detectors in similar fields as well as oil exploration and environmental remediation.

### 4.5. Other

Other selected cerium compounds also have many applications of interest. Hydrated Ce oxide is used in the production of cerium salts and cerium oxide [21]. Cerous fluoride (CeF_3_) is used in the preparation of Ce metals as well as in arc carbons to increase brilliance [21].

### 4.6. Nanoparticles: Catalysts, Glass Additives, and Fuel Additives

Cerium in its nanoparticulate form is especially important when considering applications of Ce. Nanoparticles are substances that are less than 100 nm in all three dimensions. Although the applications of compounds such as CeCl_3_ and CeBr_3_ discussed above are important, Ce compounds are best known for their uses in catalysts, fuel cells, glass (de)pigmentations, and fuel additives. All of these applications are based on the same compound: cerium dioxide (CeO_2_), or ceria. Ceria, which can be in the form of a white, pale-yellow, or brownish power depending on its purity, is by far the most widely commercially used cerium compound.

Ceria exhibits cubic crystal structure akin to a fluorite type structure. The fluorite structure of CeO_2_ allows for rapid diffusion of oxygen as a function of the number of oxygen vacancies since the oxygen atoms in the crystal are all in plane with one another. As the number of vacancies increases, the oxygen is more easily able to move around in the crystal, allowing reduction and oxidation of molecules or co-catalysts on the crystal’s surface. It is used as a co-catalyst in a number of reactions, including the water-gas shift and steam reforming of ethanol or diesel fuel into hydrogen gas and carbon dioxide. It is useful in the Fischer-Tropsch reactions and in selected oxidations [26].

Gadolinium, samarium, or platinum doped CeO_2_ is a useful material for solid oxide fuel cells due to its relatively high oxygen ion conductivity at intermediate temperatures [21]. Substituting a fraction of the CeO_2_ with these elements is a way to increase ionic conductivity and create a better electrolyte since this introduces oxygen vacancies in the crystal without adding electronic charge carriers. A large amount of oxygen vacancies within the electrolyte will form on the anode (reducing) side of the fuel cell. Some of the ceric oxide is reduced to cerous oxide under these conditions; this transformation increases the electronic conductivity of the material. The lattice constant of CeO_2_ increases under reducing conditions as well as with decreasing nanocrystal size as a result of this reduction of the cerium cation from Ce(IV) to Ce(III). This increase compensates for the formation of oxygen vacancies, resulting in an increase in the fuel cell efficiency.

Nanoparticulate CeO_2_ has a particularly high oxygen storage capacity which, when coupled with its ease of transition between trivalent and tetravalent states and its high natural abundance, makes it an excellent choice as a catalyst. Nanoparticulate CeO_2_ is most often used in catalytic converters in automobiles due to its non-stoichiometric ability to give up oxygen without decomposing. Ceria can release or take in oxygen in the exhaust stream of a combustion engine depending on its ambient partial pressure of oxygen. Through this mechanism, NO_x_ emissions are reduced and carbon monoxide is converted to carbon dioxide. Ceria is relatively inexpensive but its addition has been shown to provide substantial reductions in the amount of platinum catalyst needed for complete oxidation of NO_x_ and other harmful incomplete combustion products [26]. Fuel burns more cleanly upon addition of CeO_2_, resulting in less air pollution. It has also been characterized as a useful catalyst in systems including oxidation of volatile organic carbon [27] and toluene [28], ozonation of organic compounds [29], and water-gas shift reactions [30].

Because CeO_2_ is remarkably insoluble in water and in dilute acid [21] it is commonly used as an abrasive; the powder is used in the grinding/polishing of other materials. For many years, it was used for polishing specialized glass (telescope mirrors, for example) [31]. It is also used in heat-resistant alloy coatings and in ceramic coatings.

### 4.7. Potential Future Applications of Cerium (III) Oxides: the Ceria Cycle for Hydrogen Production and Catalysts

While CeO_2_ exhibits a particularly large wide variety of uses, another useful oxide of cerium must be mentioned as well: Ce(III) oxide. Cerium dioxide and Ce(III) oxide (or cerous oxide) are combined in an extremely useful process known as the ceria cycle. The ceria cycle serves as a pathway of great interest for hydrogen production. This thermochemical process splits water into hydrogen and oxygen and is carried out in two steps: the endothermic dissolution of CeO_2_ followed by the exothermic hydrolysis of Ce_2_O_3_. The high reactivity of Ce_2_O_3_ with steam draws the main interest in this cycle; however, a major drawback lies in the partial vaporization of CeO_2_ during the reduction step at reduced pressure. Water is the only material input to the ceria cycle and heat is the only energy input, making the ceria thermochemical cycle “a promising process for hydrogen production.” The ceria cycle is also sometimes referred to as the cerium oxide cycle [32].

## 5. Toxicity of Cerium

### 5.1. Nanoparticle Toxicity

Nanoparticle toxicity is only well-understood for a small range of nanomaterials since the field is relatively new. Nanoparticles tend to sorb more than their bulk counterparts and exhibit multiple exposure routes for biological organisms including ingestion, inhalation, absorption/adsorption onto surfaces, uptake across epithelia, and multiple other pathways. Several toxicity mechanisms have been suggested for a variety of NPs.

In general, two major considerations for NP toxicity are (1) size relative to their bulk counterparts; and (2) mechanisms often associated with capping agents. Nanoparticle toxicity characteristically exhibits size-dependent effects since their high surface to volume ratio due to the fact that a high percentage of atoms are located at the surface [33]. For a large variety of NPs including species such as NP silver, silicon dioxide, and titanium dioxide it has been demonstrated that smaller sizes (and thus greater reactivities) lead to heightened toxicity effects across biological systems. Nanoparticles tend to exhibit much higher toxicities than their bulk counterparts; this tendency is especially exaggerated in bacteria due to the NP’s higher affinity for attachment to bacterial cell walls [34] that could potentially perturb the electron transfer reaction and respiration.

As mentioned previously, NPs have much larger surface areas than their bulk counterparts and thus they also often exhibit much greater reactivity and ability to interact with biological systems. Several toxicity mechanisms have been postulated: (1) the production of ROS; (2) the release of reaction products (e.g., ionic metal(loid)s via (non)reductive dissolution); (3) photocatalytic behavior; and (4) phase transformation (e.g., sulfidation in the case of Ag(0)). A primary mechanism of NP toxicity is thought to be the ability to generate ROS such as free radicals [33]. Reactive oxygen species contain unpaired valence electrons which yield extremely high chemical reactivity and the ability to induce severe damage to cells, including DNA and RNA damage, oxidations of lipids and proteins, and enzyme (cytochrome P) deactivation. A wide array of NPs has been found to induce ROS production; among these are carbon nanotubes (CNTs), fullerenes (C_60_), and titanium dioxide NPs (TiO_2_). The release of metal ions from quantum dots (e.g., CdSe) and nanosilver can be highly toxic to bacterial cells [35,36,37] as well as organic coatings of NPs [38]. Furthermore, surface charges arising from coating/capping agents of NPs affect homo- and hetero- aggregation behavior. This could facilitate the NP interactions with cells, resulting in the oxidative stress and consequences. In general, polymers are popular capping agents along with other organic materials (e.g., citric- and oleic-acid) although the quality of capping agents is varied in different NPs [39].

### 5.2. Conflicting Views: Are CeO_2_ NPs Harmful or Beneficial?

Recent research on NP CeO_2_ has reported conflicting results regarding toxic effects: NP CeO_2_ has been reported to act as both an antioxidant and a ROS production-inducing agent across multiple biological pathways.

Many researchers have reported that CeO_2_ NPs utilize their ability to scavenge free radical species in order to act as an antioxidant. Ceria NPs have been reported by multiple researchers to act as a mimetic of superoxide dismutase, the main catalyst that reduces the production of and damage caused by ROS to mammalian cells by transforming superoxide into oxygen and hydrogen peroxide. They have been shown to inhibit cellular aging [40] and have even demonstrated the ability to increase the lifespan of brain cell cultures [41]. Their properties have been implied in the prevention of retinal disorders leading to blindness [42]. However, other research seems to tell a opposed story about CeO_2_ NPs. Multiple researchers report strikingly opposite effects. Ceria NPs have been reported to induce and catalyze the generation of ROS across multiple biological systems. Nanoceria has been reported to lead to decreased lifespans in roundworms [43], cause liver damage in rats [44], and exhibit moderate toxicity to various human muscle tissues including lung tissue [45]. Although the recent research indicated that the differences in the particle size of CeO_2_ NPs and the ratio of Ce^3+^/Ce^4+^ content might have led to these apparent antagonist behaviors of nano ceria [6,46], more research evidence is needed before any conclusions can be drawn about the mechanisms of harmful and or beneficial effects of CeO_2_ NPs.

## 6. Environment Fate of CeO_2_ Nanoparticles

**Figure 1:**
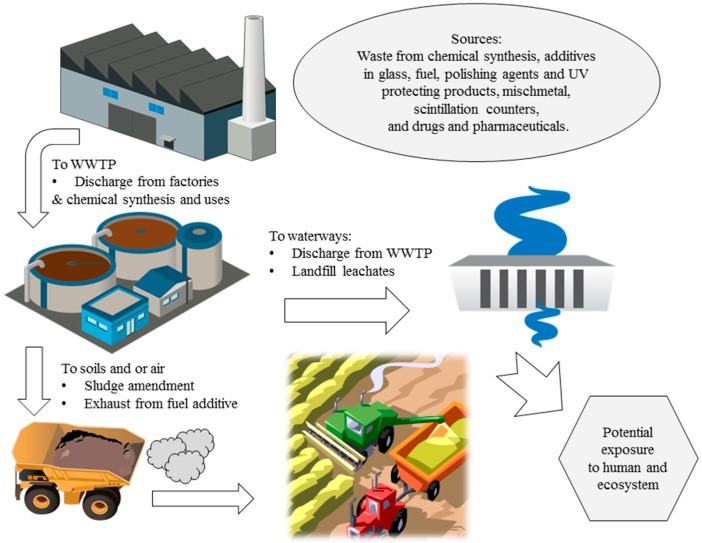
Environmental fate of cerium based consumer and industrial products including CeO_2_ nanoparticles. Illustrations of water treatment plant, factory and water discharge are from integration and application network, [53].

The environmental fate of CeO_2_ NPs is dependent on its applications and uses. A schematic illustration of environmental fate of cerium based industrial and consumer products are shown in Figure 1. As previously stated, the use of cerium as a diesel fuel additive coupled with a particulate filter drastically decreases particulate matter emissions in automobile exhaust, up to 90% by weight [47]. Some Ce escaping in the emissions could accumulate in soils. Exhaust particulate matter exists as very fine particles, normally < 1 μm, leading to extremely diffuse pollution through air exposure [48]. Cerium is also introduced to landfills as solid wastes of electronics, soils via recycling of sewage sludge, and the aquatic environment via wastewater discharge from ceramic manufacturing plants [49].

When particles are not efficiently removed from wastewater streams, they can become a long-term problem for the environment. A study by Limbach, *et al.* (2008) has shown that, for a model wastewater treatment plant, a significant fraction of CeO_2_ NPs were able to escape the clearing system and avoid adsorption to clearing sludge [50]. The surface charge of the NPs was found to play a key role in the stabilization against clearance, leading to detectable concentrations of CeO_2_ NPs up to 6 wt % in the model plant’s exit stream [50].

At the wastewater treatment plant, about 95% of CeO_2_ NPs undergo biosolid/sludge accumulation in wastewater treatment plants. Thus CeO_2_ discharge into the aquatic environment is fairly limited as compared to accumulation levels in the terrestrial environment including landfills [50]. In general, engineered NP partitioning is the most significant in wastewater treatment plant biosolids/sludge, it follows that Ce pollution could occur after the amendment of agricultural soil via application of wastewater biosolids. As in the case of many other metal NPs such as TiO_2_, ZnO, and Ag NPs [51], it is believed that biosolids application to agricultural soils serves as one of the largest environmental exposure pathways for CeO_2_ NPs [52].

Unfortunately, due to the relativeness newness of discovery of CeO_2_ NP chemistry as a field, the fate and transport of NPs in general are not still well-understood. Much work has been done with TiO_2_ and silver NPs but most NPs have not been studied as extensively. Nanoparticulate CeO_2_, while greatly useful for a wide variety of applications, is not without the risks and hazards associated with NPs in general. The toxicity of cerium NPs and cerium in general is discussed in depth in Section 5.

Cerium oxide NPs are of great interest biologically for many reasons, including their many starkly contrasting toxicity effects in different conditions. As previously mentioned, increased Ce(III):Ce(IV) ratios result in increased SOD mimetic activity and thus further inactivation of superoxide free radicals [6]. Unlike SOD, CeO_2_ NPs can directly react with hydroxyl radicals [54] as well as superoxide radicals in order to inactivate both short-lived and stable radicals [55]. A study by Ivanov, *et al.* showed that the rate of inactivation of stable radicals by CeO_2_ NPs is dependent directly upon particle size and that smaller particle sizes result in increased rates of radical inactivation [56]. Discussed below are effects of CeO_2_ NP exposure to biological organisms including plants, bacteria/aquatic organisms, and mammals, as well as the recorded and possible effects of these exposures.

### 6.1. Plants

Once in the soil, cerium NPs may be absorbed into vegetation or contaminate water. Cerium has been shown to be likely to enter the produce food chain through take-up by alfalfa, corn, tomato, cucumber, and soy [57,58]. Nanoparticles tend to accumulate on root surface of plants, decreasing hydraulic conductivity and often inhibiting root growth [59]. Nanoceria has been reported to significantly reduce germination rates in corn, tomato, and cucumber; the same study reported enhanced root growth in cucumber and corn species after introduction of CeO_2_ NPs, but reduced root growth in tomato and alfalfa plants. Shoot elongation was also found to be enhanced in all four species [57]. Another study displayed differing effects, finding that CeO_2_ NPs did not affect root growth in radish, tomato, lettuce, wheat, rape, cabbage, and cucumber plants [60]. Concentration-dependent absorption by plant roots has been observed, but most of the NPs are instead adsorbed to the surface of the root; entry into the roots is limited and very difficult [61]. In a recent spectroscopic investigation, the fate of CeO_2_ NPs in soybean plants was investigated in fully grown plant tissues [62]. The X-ray absorption spectroscopy studies showed that Ce remained mostly as Ce(IV)O_2_ NPs within the plant (pods and tissues), and a small fraction of Ce(IV) was reduced to Ce(III).

Priester and co-workers (2012) recently examined the effect of CeO_2_ NPs on soybean [62]. The researchers found an interesting dose-response relationship. Plants with low CeO_2_ NPs (100 mg/kg) grew slowly and had reduced leaf cover, whereas plants grown with high CeO_2_ NPs (1000 mg/kg) yielded less soybean biomass. They also observed the shutdown of nitrogen fixation in root nodules when plants were exposed to high CeO_2_ concentrations.

Cerium dioxide NPs have also been shown to protect anthocyanins in grapes from ROS by inhibiting oxidative degradation in neutral and alkaline solutions. However, in acidic solutions, no inhibitory effects were observed after exposure of the anthocyanins to NP CeO_2_ [63]. Similar pH-dependent effects have been demonstrated across a variety of plants and other organisms.

Cerium dioxide NPs have also shown translocation ability; that is, the capability to cross biological barriers such as the blood-brain barrier. When applied to maize leaves, CeO_2_ NPs was demonstrated to either adsorb to the leaf surface in agglomerated form (larger particles) or penetrate the leaf and become incorporated into the leaf structure (smaller particles). However, in plants cultivated after airborne exposure to CeO_2_ NPs, newly grown leaves exhibited no translocation. This lack of evidence for translocation is important since maize is a major agricultural crop and knowledge of NP ability to translocate into newly grown, non-exposed plants is needed. Nanoparticles most commonly enter plants via root uptake or air exchange into the leaves. Nanoparticles larger than 1.1 µm were found by the study to be unable to penetrate the leaf surface and enter the leaves [64].

### 6.2. Bacteria and Algae

Nanoparticle effects on both Gram-positive and Gram-negative bacteria have been studied extensively. Cerium dioxide NPs were shown to exhibit size-dependent and concentration-dependent inhibition of both *Escherichia coli* and *Bacillus subtilis*; it is suggested that the NPs do not penetrate the bacterial cells, but rather adsorb to them, inducing deleterious effects. The same study found that CeO_2_ NPs induced no inhibitory effects on the growth of *Shewanella oneidensis*, demonstrating that bacterial responses to NPs and interactions between the two vary greatly among bacterial species [65].

Another study on cytotoxicity of CeO_2_ NPs to Gram-negative *E. coli* also reported that the NPs were adsorbed in large amounts on the outer membrane [66]. The NPs are positively charged at neutral pH values, meaning they are subject to a strong electrostatic attractive force toward bacterial outer membranes such as that of *E. coli*. Close contact was reported between the NPs and *E. coli*, indicating that oxidative response may have been responsible for the observed toxicity, which was significant after the adsorption of the NPs and their reduction from Ce(IV) to Ce(III). The survival rate of *E. coli* was reduced to half for a 5 mg/L exposure to CeO_2_ [66]. This study and other investigations have found that cytotoxicity of CeO_2_ NPs for *E. coli* can be reversed by changing the exposure media, indicating that surface charge density is largely responsible for NP CeO_2_ cytotoxic effects [67].

Zeyons and co-workers (2009) also observed that toxicity requires direct contact between CeO_2_ NPs and cells of *Synechocystis* PCC6803 cyanobacteria and the RR1 strain of E. coli [68].

More recently, Dahle and Arai investigated a subchronic toxicity study of CeO_2_ NPs on soil-denitrification process as a function of particle size (33 and 78 nm), total Ce concentration (50–500 mg/L), and speciation [Ce(IV) *vs.* Ce(III)] [69]. Soluble Ce(III) was far more toxic than Ce(IV)O_2_ NPs when an equal total concentration of Ce was evaluated. Particle size-dependent toxicity, species-dependent toxicity, and concentration-dependent toxicity were all observed in this study for both the steady-state and the kinetic evaluations. They suggested that changes in physicochemical properties of Ce(IV)O_2_ NPs in soil solution might be important in assessing the environmental fate and toxicity of NPs in aquatic and terrestrial environments.

In a study on the effect of NPs on microbial communities in wastewater treatment plants, it was found that CeO_2_ NPs exhibited stronger inhibitory effects on biogas production by the microbes than TiO_2_, gold, or silver NPs [70]. After release into the waterway, CeO_2_ NPs are far more likely to partition into aquatic plants and sediment than to stay in the water, but some aquatic species such as algae and fish are still at risk and the possible toxicity and effects of CeO_2_ NP uptake in these organisms must be investigated [71]. When CeO_2_ NPs do remain in the sediment, concentrations tend to be locally high due to the adsorption and aggregation tendencies of NPs, as stated earlier.

Rodea-Palomares and co-workers observed the species specific aquatic toxicity to CeO_2_ NPs [72]. The aquatic cyanobacteria *Anabaena* CPB4337 exhibited toxicity to CeO_2_ NPs values ranging from 0.27 to 6.3 mg/L while the aquatic green alga *Pseudokirchneriella subcapitata* exhibited values from 2.4 to 29.6 mg/L. A few studies reported that the growth inhibition of *P. subcapitata by* CeO_2_ NPs was at 10.3 mg/L [73] and 7.6 mg/L [74]. Cerium dioxide NPs were shown to cause membrane disruption and severe damage to the cells of both organisms though uptake of the NPs by the cells was not indicated. As in the case of *E. coli* and *B. subtilis* mentioned above, the *Anabaena* cells became coated with adsorbed CeO_2_ NPs. It is suspected that cell wall and membrane disruption was likely the cause of cell damage rather than oxidative activity [72]. From these and other similar studies, it remains unclear whether nanoceria toxicity can be largely attributed to cellular adsorption or if uptake of the particles lends to intracellular effects, especially because negatively charged particles should be repelled by the large negatively charged domains of cell membranes. Importantly, it has been demonstrated that CeO_2_ NP toxicity in *P. subcapitata* is also influenced by pH, ionic strength and amounts of natural organic matter. Increasing ionic strength levels result in increased CeO_2_ NP aggregation, while higher levels of natural organic matter sharply lowered average NP aggregate sizes, severely decreasing aggregation from particle sizes of ~4500 nm to sizes of 100–200 nm. Adsorption of the NPs to natural organic matter reduced their bioavailability and thus lowering the toxicity of CeO_2_ NPs to *P. subcapitata* [75]. However, Mariner and co-workers (2013) reported that changes in agglomeration state of CeO_2_ NPs did not significantly inhibit the growth of *P. subcapitata* [76]. The EC_50_ values were 5.6, 4.1 and 6.2 mg/L, after exposure to non-aged (non-aggregated), 3 and 30 days aged aggregated NP suspensions, respectively.

### 6.3. Invertebrates

*Caenorhabditis elegans* is a commonly-studied soil nematode that serves as an important aquatic indicator. Ceria NPs have been shown to induce size-dependent toxicity effect on the survival and fertility of *C. elegans*; while all CeO_2_ NP exposure sizes in one study led to decreased reproductive ability and increased fatality, the smaller NPs had a markedly more toxic effect than the larger NPs. This effect was also observed for TiO_2_ NPs [77]. Another study on nanoceria exposure in *C. elegans* indicated that CeO_2_ NPs could lead to reduced lifespans via catalysis of ROS generation and subsequent oxidative damage. The study observed a 12% reduction in mean lifespan even at the lowest CeO_2_ NPs exposure level of 1 nM [43].

While an early study did not report the acute toxicity of CeO_2_ NPs to two crustaceans *Daphnia magna* and *Thamnocephalus platyurus*, even ranging up to 5000 mg/L [76], few recent studies showed some toxicological effects of CeO_2_ NP to *Daphnia. Artells* and co-workers (2013) evaluated the effects of CeO_2_ NPs ([CeO_2_]_total_ = 1–100 mg/L, exposure time: 48 h) on the survival and the swimming performance of *Daphnia similis* and *Daphnia pulex* [78]. EC_50_ for *D. similis* (0.26 mg/L) was 350 times more sensitive than *D. pulex*. They suggested that the interspecific toxic effects are probably due to morphological variations such as the presence of reliefs on the cuticle and a longer distal spine in *D. similis* that effectively trap NP aggregates, yielding in a greater sorption of NP in *D. similis*. Swimming velocities were significantly affected both species. Biodistribution of CeO_2_ NP in *Daphnia pulex* was also investigated during a molting stage [79]. A CeO_2_ NP uptake was increased by a factor of three when algae were present during the NP exposure. Although NPs were localized in the gut content, the depuration was not efficient to remove the NPs. Interestingly, shedding of the chitinous exoskeleton (every 59 ± 21 h with growth rates about 1.1 or 1.8 μm per stage) was the crucial mechanism controlling the release of NPs regardless of the feeding regime during exposure.

Using an integrated multibiomarker approach, short-term (<100 h) toxicological effects of CeO_2_ NPs on two freshwater invertebrates; the amphipod *Gammarus roeseli* and the bivalve *Dreissena polymorpha*, were investigated [80]. Under the environmentally realistic concentrations (1–100 µg/L), *G. roeseli* accumulated more Ce than *D. polymorpha,* though no strong adverse effects were observed on G. roeseli. However, the exposure led to decreases in the size of the lysosomal system, catalase activity and lipoperoxidation in mussel digestive glands.

Conway *et al.* studied the effects of short-term (5 wk) exposure of CeO_2_ NPs in a marine mussel, *Mytilus galloprovincialis* [81]. The direct and the indirect exposure (NP sorbed phytoplankton) exposure of CeO_2_ NPs (CeO_2_: 0–3 mg/L) did not make any significantly different in the [Ce]_total_ accumulation in dry mussel tissue or pseudofeces. Although ~99% of CeO_2_ was excreted, the depuration rates decreased with increasing time in groups exposed to CeO_2_ directly, indicating stress.

### 6.4. Vertebrates

Larger-scale organisms should also be considered when evaluating the ecotoxicological effects of CeO_2_ NPs. In a study exposing multiple nanoscale metal oxides (zinc oxide, CeO_2_, and titanium dioxide) to zebrafish (*Danio rerio*) and rainbow trout (*Oncorhynchus mykiss*) both via water and via diet, the oxide NPs were largely not found to be able to penetrate the fish cell barriers. The zinc, cerium, and titanium content was measured in the gill, liver, skin, brain, but, blood, and kidneys of the two fish species and only two areas of significant NP uptake were observed. Ceria NP uptake was discovered in the liver of the zebrafish exposed via water and ionic titanium was detected in the gut of the trout exposed via diet. It is likely that this inability of the metal oxide NPs, including CeO_2_, was due to limited bioavailability caused by accumulation of large aggregates (up to 3 μm) in the aqueous exposure route [82].

Mammals are among the lesser-studied organisms for CeO_2_ NP toxicity effects; unlike the widespread variety of plants, bacteria, and aquatic organisms, the only mammal cell lines that have been evaluated for CeO_2_ NP toxicity currently include those of rodents and humans. Cerium dioxide NPs have been demonstrated to be neuroprotective in mouse hippocampal brain slices, indicating that the NPs could be useful as therapeutic agents for strokes due to reduced ischemic cell death [83]. Another study of rodent cell line HT22 verified that CeO_2_ NPs (as well as yttrium NPs) prevent oxidative stress to cells and are neuroprotective [84]. However, it has also been shown that a single CeO_2_ NP exposure leads to reduced liver weight, hepatocyte enlargement, reduced albumin levels, and diminished sodium-potassium ratios in male Sprague-Dawley rats [44].

Cerium dioxide NPs have demonstrated a range of activity in human cells as well. Unlike bacterial and algal cells, effective uptake of CeO_2_ NPs by human cells has been demonstrated repeatedly. It has been shown that CeO_2_ NPs modulate the brain-derived neurotrophic factor (BDNF) pathway in human Alzheimer’s disease models in order to trigger neuronal survival [85]. The NPs have been shown to exhibit toxicity to bronchial epithelial cells due to ROS production [86], although another study showing the same results for bronchial epithelial cells also found no toxic effects for human brain cells and rat cardiomyocytes [45]. Another study exhibited low to moderate toxicity of CeO_2_ NPs to human alveolar epithelial and macrophage cell lines [87]. In a study comparing CeO_2_ to Al_2_O_3_ and ZnO NPs, CeO_2_ NPs were found to induce membrane damage and inhibit colony formation to differing degrees across human lung epithelial cells, carcinoma cells, and normal cell lines. The CeO_2_ NPs did not demonstrate notable adverse effects on cell proliferation and viability; they were shown to be much less toxic than ZnO in terms of cell viability, proliferation, colony formation, and membrane integrity, but more toxic than Al_2_O_3_ even after long-term exposure [88].

As previously stated, the results of studies across similar cell lines are not always in agreement. Cerium dioxide NPs have been reported to both induce and prevent oxidative stress in a variety of organisms. Studies have shown that existing levels of CeO_2_ in the environment are far “lower than the estimated no effect dose for chronic human exposure” [45], leading to the assertion by some that CeO_2_ NP, along with a variety of other NPs, may not exhibit any environmental effects at all [75]. However, until the ecotoxicological effects of CeO_2_ NPs are well-understood, studies must continue to investigate the generation, transport, uptake, and toxicity mechanisms of these potentially harmful materials.

## 7. Conclusions

A cerium compound of particular interest is nanoparticulate ceria, also known as cerium oxide (CeO_2_). By far, CeO_2_ NPs are the most widely commercially used cerium compound for electronics, automotive, and solar panels for energy. As the production of CeO_2_ NP increases, so does the usage in the industry and consumer markets. The recent study by Keller and Lazareva (2013) predicted more than 60%–86% of engineered NPs including CeO_2_ NPs will end up in landfills and soils, and the pathway of WWTP effluent and biosolids to the soil-water environment is fairly limited [49]. The estimated concentrations of CeO_2_ NPs in WWTP in the San Francisco Bay (SFB) area are 10^−2^ to 1 µg/L in effluent and 1 to 10 µg/kg in dry biosolids. In other study by Gottschalk *et al.* (2013) [89], even much smaller concentration of CeO_2_ NPs in WWTP effluent and biosolid are reported (0.5 × 10^−4^ µg/L and 10^−6^ mg/kg, respectively). While there are needs in improving the detection and quantification of CeO_2_ NPs in different environmental media, it is imminent that organisms in ecosystem will be exposed to these low concentrations (micro gram per liter or kilogram).

In the last decade, scientific community has begun to uncover the effect of engineered NPs on human and ecosystem health through environmental chemistry and toxicology research in plants, bacteria, aquatic organisms, and mammals. However, the research findings are still difficult to extrapolate to natural aquatic and terrestrial systems since (1) some toxicological data are contradicting (e.g., CeO_2_ as antioxidant or reactive oxygen species production-inducing agent) [40,41,44,45]; (2) inconsistent characterization of NPs (e.g., ratio of Ce^3+^ and Ce^4+^); and (3) the results are not often generated under the environmentally relevant geochemical conditions (e.g., concentration, chemical species) that directly affect the NP reactivity (e.g., solubility and complexation with inorganic and organic ligands) and toxicity. In understanding the geochemical fate and toxicity of CeO_2_ NPs, it might be important in considering the relevant geochemical conditions (pH and common ligands, phosphate) [90] in the future toxicological and environmental chemistry investigations.
